# Exploring Adverse Event Associations of Predicted PXR Agonists Using the FAERS Database

**DOI:** 10.3390/ijms26157630

**Published:** 2025-08-06

**Authors:** Saki Yamada, Yoshihiro Uesawa

**Affiliations:** Department of Medical Molecular Informatics, Meiji Pharmaceutical University, Tokyo 204-8588, Japan

**Keywords:** pregnane X receptor, machine learning, cardiac disorders, FAERS database, adverse event

## Abstract

Pregnane X receptor (PXR) is an important nuclear receptor that regulates diverse physiological functions, including drug metabolism. Although PXR activation is potentially involved in adverse events, the full scope of its impact has yet to be elucidated. In this study, we developed a machine learning model to predict the activity of PXR agonists and applied the model to drugs listed in the US Food and Drug Administration Adverse Event Reporting System database. Analysis of the predicted agonist–active drug interactions and adverse event reports revealed statistically significant risks (lnROR > 1 and −log*p* > 1.3) for multiple cardiac disorders. These findings suggest that PXR activity is involved in cardiovascular adverse effects and may contribute to drug safety through the early identification of risks.

## 1. Introduction

Pregnane X receptor (PXR) is a ligand-dependent transcription factor belonging to the nuclear receptor superfamily [1]. PXR is highly expressed in the liver and small intestine [2]. PXR was initially identified as an important regulator of endogenous substance, drug, and foreign body metabolism [3], but subsequent studies revealed its broader functions in important physiological processes such as bile acid detoxification and excretion, glucose and lipid homeostasis, cholesterol metabolism, inflammation, bone metabolism, bilirubin clearance, oxidative stress, and cancer [4,5,6,7,8,9,10,11]. This receptor can cause adverse effects through excessive activation or interactions with specific drugs, making it a critically important molecular target in drug–drug interactions and toxicity [12,13]. In fact, PXR activation is involved in the manifestation of toxicity and metabolic disturbances, including enhanced hepatotoxicity, exacerbation of liver injury, and glucose metabolism abnormalities mediated by the HNF4α–GLUT2 pathway [14,15,16,17,18,19]. However, all of these findings were generated in studies limited to individual drugs and disease models, and the overall picture of side effects associated with PXR agonist activity is not fully understood.

The US Food and Drug Administration (FDA) Adverse Event Reporting System (FAERS) collects drug-related adverse event information [20]. Using this database, it is possible to detect trends in adverse events related to PXR agonist activity and identify new safety signals, thereby improving drug safety. However, because only a limited number of drugs have been experimentally assessed for PXR agonist activity, no comprehensive analysis of drugs and side effects has been conducted. Therefore, we constructed a predictive model based on compound data related to PXR agonists provided by the Toxicology in the 21st Century (Tox21) program and applied it to drugs listed in the FAERS database to analyze trends in adverse events related to PXR agonist activity in an effort to clarify the impact of PXR activation on drug safety.

## 2. Results

### 2.1. Results of the PXR Agonist Prediction Model

PXR agonist data were obtained from the Tox21 database provided by PubChem (https://pubchem.ncbi.nlm.nih.gov/source/824, accessed on 13 June 2025). After optimizing and preprocessing the obtained SMILES structures using Molecular Operating Environment (MOE) version 2022.02 (Chemical Computing Group, Montreal, Canada), duplicate compounds were removed. Furthermore, after excluding complete correlations, 336 molecular descriptors were calculated from these structures and used as explanatory variables for the machine learning model. Using the calculated molecular descriptors as explanatory variables and the presence or absence of PXR agonist activity (binary) as the target variable, a classification model was constructed using Light Gradient Boosting Machine (LightGBM) version 4.6.0. In external validation of the model, the sensitivity, specificity, balanced accuracy, Matthews correlation coefficient (MCC), and area under the curve (AUC) were 0.8386, 0.8069, 0.8228, 0.5630, and 0.8962, respectively, and the optimal cutoff was 0.2194.

**Figure 1:**
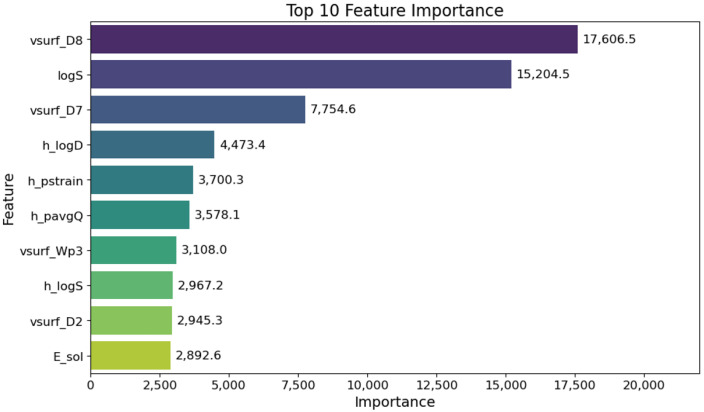
Top 10 most important features in the PXR agonist prediction model using LightGBM. The values were calculated according to the gain obtained from the model.

On feature importance analysis, molecular descriptors with particularly high contributions in the PXR agonist prediction model included vsurf_D and vsurf_Wp, which are related to hydrophobic and polar volumes, respectively. Other features with strong contributions included logS and h_logS representing solubility, h_logD representing the partition coefficient, h_pstrain representing strain energy, h_pavgQ representing the average total charge (pH 7), and E_sol representing solvent energy (Figure 1). These descriptors are believed to reflect structural and physicochemical features related to PXR agonist activity.

### 2.2. Predictive Model Application to Drugs Listed in the FAERS Database

The constructed PXR agonist prediction model was applied to 5523 drugs included in the FAERS Drug Information (DRUG) table for which the SMILES notation was available. In total, 1322 drugs were predicted to have PXR agonist activity (Supplementary Table S1). PXR agonist activity was experimentally confirmed in the Tox21 database for some drugs. These drugs were treated as having a prediction probability of 1 in the analysis, as they were experimentally confirmed PXR agonists. Among them, 16 compounds also showed model-predicted probabilities greater than 0.95: apigenin, butoconazole, daidzein, danazol, dichlorophen, econazole, genistein, interferon alpha-2B, isoconazole, omoconazole, oxiconazole, prazepam, resveratrol, sulconazole, tioconazole, and tolnaftate.

**Figure 2:**
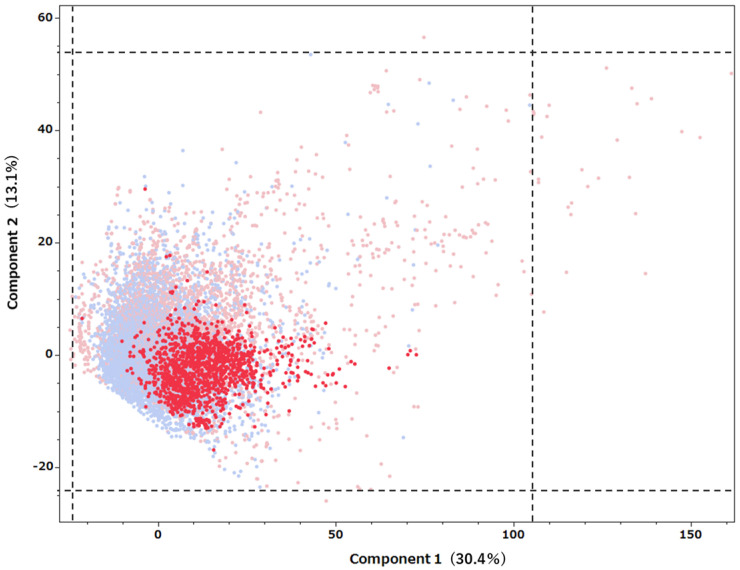
Comparison of the structural distributions of compounds listed in Tox21 and FAERS databases using PCA. The graph presents the relationship between principal component 1 and principal component 2. Tox21 compounds (blue) and FAERS drugs (red) are presented, with dark red dots indicating drugs determined to have PXR agonist activity by the prediction model. The dotted frame in the figure presents the distribution range of the main components of the compounds included in the Tox21 database, which was used as a guideline for the applicability domain.

To visually evaluate the validity of the applicability domain of the predictive model, principal component analysis (PCA) was performed on compounds listed in the Tox21 database, and the first and second principal components were extracted. Using the calculated formulas for the principal components obtained in this process, the drugs listed in the FAERS database were also projected onto the same principal component space (Figure 2). The contributions of principal components 1 and 2 were 30.1% and 13.1%, respectively. Many of the predicted active drugs were distributed in the PCA space in the area overlapping the compounds in the Tox21 database, indicating that the predictions were made within the applicability domain.

### 2.3. Relationships Between Drugs with Predicted PXR Agonist Activity and Adverse Events

**Figure 3:**
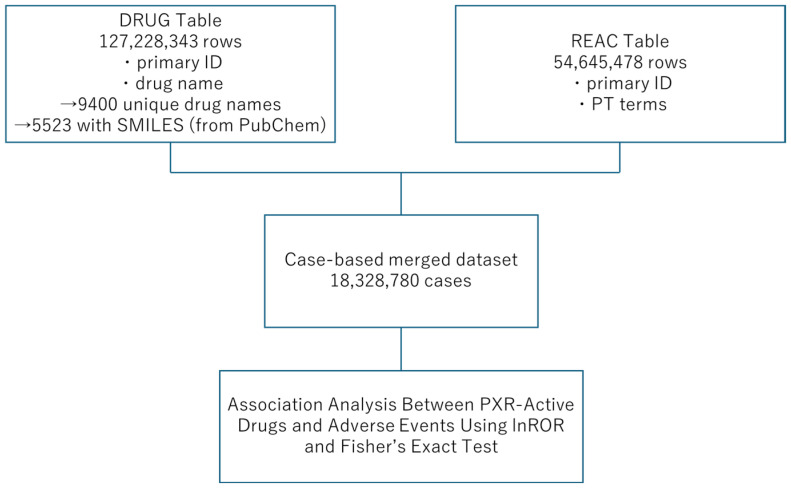
Flowchart for creating tables for analysis.

**Figure 4:**
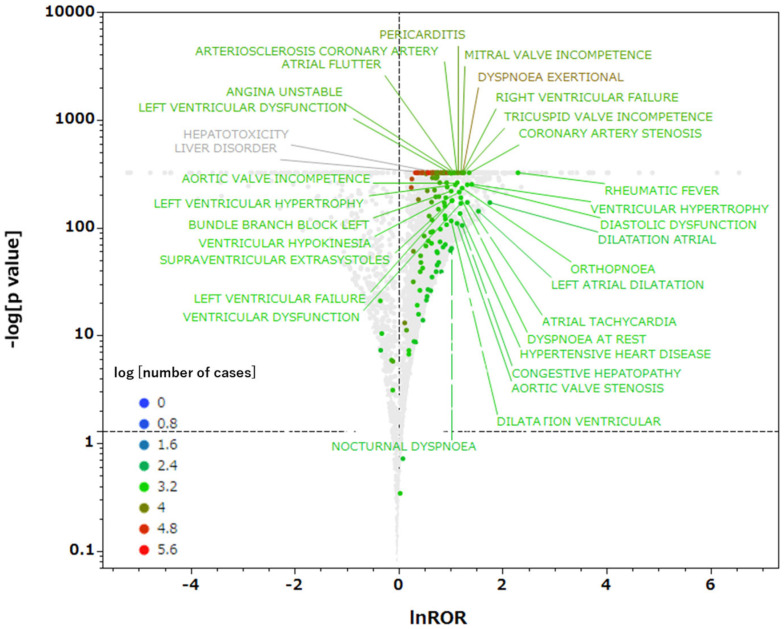
Volcano plot presenting the associations between drugs with predicted PXR agonist activity and adverse events. The vertical axis presents statistical significance based on Fisher’s exact probability test (−log*p*), and the horizontal axis presents the adverse event risk of PXR agonists (lnROR). Each point represents an adverse event term, and the color of the points denotes the number of reports for that event. This plot focuses on adverse events with at least 1000 reports. Terms corresponding to “cardiac disorders” in the MedDRA SOC classification are displayed in color, whereas terms belonging to other SOCs are presented in gray. Within the figure, terms labeled with their names are those for cardiac disorders deemed statistically significant (lnROR > 1 and −log*p* > 1.3). Additionally, “Hepatotoxicity” and “Liver disorder” are labeled in gray.

First, we combined the FAERS DRUG table (127,228,343 rows) and Adverse Reaction (REAC) table (54,645,478 rows) to create a case-based database (Figure 3). In this database, each case report was counted only once, and thus, the analysis examined whether both the drug and adverse reaction were present in the case for each drug–adverse reaction combination. The analysis involved extracting cases containing drugs with predicted PXR agonist activity from the total number of cases (18,328,780 cases). Adverse events with 1000 or more reported cases were extracted and analyzed to examine the correlations between medications with predicted PXR agonist activity and reported adverse events. In total, 323 adverse event terms were examined (Supplementary Table S2), and when these were controlled using MedDRA system organ class (SOC) categories, cardiac disorders were significantly more common than other terms (Table 1). Previous studies reported that PXR activation enhances hepatotoxicity and worsens liver damage [14,15,16]. However, in this analysis, lnROR was 0.756 for hepatotoxicity and 0.562 for liver disorder, respectively, both of which were lower than 1 (Figure 4). Although the −log*p* values for both were statistically significant at ≥323, PXR agonists were not confirmed to be more likely to induce these adverse events. Additionally, the proportion of statistically significant terms classified under the SOC category “Hepatobiliary disorders” was 1.22% of the total within that SOC category, which was not particularly high compared with the values for other major SOC categories. Therefore, the results did not conclusively indicate that PXR agonists cause clinically significant liver damage. Other reports also mentioned the involvement of PXR in metabolic abnormalities [17], but the proportion of statistically significant terms classified under “Metabolism and nutrition disorders” was 1.74%, which was not significantly higher than the values for other major SOC categories (Table 1).

## 3. Discussion

### 3.1. Applicability of the PXR Agonist Prediction Model to Drugs Listed in FAERS

We constructed a machine learning model based on 336 molecular descriptors using data on PXR agonists registered in the Tox21 database and achieved high external validation performance. Many of the drugs predicted by the model exhibiting structurally similar PCA distributions as known PXR agonists, supporting the external validity of the model.

The features extracted as important by this model included vsurf series descriptors, such as vsurf_D8, vsurf_D7, and vsurf_Wp3, as well as physicochemical descriptors related to solubility, such as logS and h_logD. These descriptors reflect the three-dimensional (3D) surface characteristics and hydrophobic distribution of molecules, as well as their behavior in solvents, suggesting compatibility with the large, flexible hydrophobic pockets characteristic of the PXR ligand-binding domain [21]. The effectiveness of these 3D structure-based descriptors was consistent with that of previous studies, demonstrating that vsurf series descriptors are important features for predicting the adverse effects of PXR agonists [22].

Furthermore, PXR agonist activity has been experimentally confirmed in the Tox21 database, with 16 agents achieving a predicted probability of 0.95 or higher. In particular, flavonoids such as genistein, daidzein, and apigenin were previously reported to exhibit PXR agonist activity or similar activity [23,24]. Furthermore, imidazole-based antifungal agents, such as oxiconazole and econazole, induce PXR-related drug metabolism enzymes [25]. However, certain imidazole antifungals were found to inhibit PXR transcriptional activity, and their pharmacological actions exhibit drug-specific variability. Resveratrol is described in the literature as a PXR antagonist, with numerous reports indicating that it inhibits PXR activation [26]. However, ligand assay results suggest that it is a “weak agonist” [27], indicating that the effects of resveratrol on PXR are ambiguous. Therefore, the classification of resveratrol as an agonist in the predictive model of this study can be interpreted to reflect structural characteristics providing binding affinity for PXR and the potential to induce activation in a condition-dependent manner.

These results suggest that this model represents a reliable tool for analyzing the PXR-mediated risk of adverse events.

### 3.2. Relationships Between Drugs with Predicted PXR Agonist Activity and Adverse Events

The volcano plot generated in this study suggested that drugs predicted to have PXR agonist activity are more likely to be associated with terms related to cardiac disorders than other adverse event terms, suggesting that PXR activation potentially affects cardiac homeostasis. Previous studies also suggested that PXR activation can alter lipid metabolism and induce cardiovascular disease risk factors. For example, Helsley et al. reported that PXR activation by the HIV drug amprenavir and environmental chemicals induces the expression of genes related to cholesterol absorption in the intestine, leading to increased LDL cholesterol accumulation and atherosclerosis progression [28]. Sui et al. found that PXR activation by environmental chemicals promotes CD36 expression in macrophages and contributes to the development of atherosclerosis through foam cell formation [29]. Furthermore, other studies revealed that 4β-hydroxycholesterol, a marker of PXR activity, is associated with factors that increase the risk of heart disease, such as obesity, hypertension, and impaired glucose metabolism [30], suggesting that PXR activation is a background factor in cardiac disorders. Regarding liver toxicity and liver disorders associated with PXR, although the −log*p* values exhibited high statistical significance, the lnROR values were lower than 1, and they were not considered adverse events induced by PXR agonists. In fact, PXR activation can exert hepatoprotective effects in some cases [31], and the direction of risk is believed to vary depending on the drug, disease state, and PXR activation pathway. Additionally, metabolic and nutritional disorders did not have a stronger relationship with PXR than did cardiac disorders. Although previous studies reported that PXR regulates various metabolic pathways, including lipid metabolism, bile acid metabolism, and glucose homeostasis [7,8], these effects do not necessarily manifest as adverse events. These findings are expected to provide important insights into the pathophysiology associated with PXR agonist activity.

### 3.3. Limitations

This study had several limitations, and caution should be exercised when interpreting and generalizing the results. First, the construction of machine learning models using quantitative structure–activity relationships might be biased toward specific structural distributions, limiting their predictive performance (generalizability) for unknown structurally different compounds. Additionally, some molecular descriptors are believed to be highly correlated, which could affect the interpretability and reproducibility of the model [32]. Second, the activity data in Tox21 are based on in vitro assays, and the direct correlation with in vivo pharmacological activity or actual adverse effects is unclear. This limitation affects the clinical significance of the predictive results. Third, this model targeted 5323 drugs for which the SMILES notation was available, and other drugs were excluded from the analysis. Therefore, the impact of compounds excluded from the analysis on the overall evaluation is unknown. Fourth, the spontaneous reporting database only records cases in which adverse drug events (ADEs) occurred, and it does not include information on all patients who used the drug [33]. Therefore, it is not possible to directly calculate the incidence rate of ADEs, and indicators such as the reported odds ratio (ROR) must be used for risk assessment, which could introduce bias. Fifth, the existence of bias in reporting must be noted. Some ADEs are not reported and, in particular, mild ADEs and new risks are often overlooked. Conversely, severe ADEs and already known risks tend to be overreported, leading to bias in the results [33,34]. Finally, in cases in which multiple drugs are used concomitantly, identifying which drug causes the ADE is difficult [33,35]. In this study, we included “primary suspect drugs” and “secondary suspect drugs” together with concomitant drugs and drug interactions in the analysis; however, this could result in false positives (FPs). Therefore, it is not possible to verify the existence of a causal relationship between drugs and adverse events, and we anticipate that future studies will provide insights that consider these factors.

## 4. Materials and Methods

### 4.1. PXR Agonist Database

To perform an inclusive analysis of pharmaceuticals with PXR agonist activity, data on PXR agonists were obtained from the Tox21 database (AID: 1347033; https://pubchem.ncbi.nlm.nih.gov/bioassay/1347033, accessed on 13 June 2025), a large-scale toxicity database based on high-throughput screening developed to perform toxicity evaluation of chemical substances in a highly efficient and predictive manner [36]. We preprocessed the data using MOE together with the SMILES and activity scores of the compounds in the database. MOE is an integrated chemical information processing software that includes molecular structure optimization, descriptor calculation, and molecular dynamics simulation [37]. For preprocessing, we performed deionization to remove salt from the molecules; protonation to add protons (H^+^) to the molecules; and 3D modeling to reproduce their structures under standard pH conditions, assignment of partial charges, and 3D modeling. Furthermore, we calculated 336 molecular descriptor types, which served as input for the machine learning model. The number of compounds was reduced from 9667 to 7237 after removing duplicate SMILES notations, and the activity score was taken as the median for each compound.

### 4.2. Creation of the PXR Agonist Prediction Model

A classification model was built to predict PXR agonist activity using LightGBM. This leaf-wise growth algorithm incorporates gradient-based one-side sampling, which prioritizes samples with high importance to achieve fast and scalable gradient boosting, and exclusive feature bundling, which groups mutually exclusive features [38]. The explanatory variables comprised 336 molecular descriptors calculated using MOE, and the target variable was the presence or absence of PXR agonist activity. An activity score of 40 or higher denoted “active” (label: 1), and a score lower than 40 indicated “inactive” (label: 0). Descriptors were checked for correlations, and those with perfect correlation were excluded. No explicit feature selection was performed for other features, as LightGBM automatically selects and evaluates them internally. LightGBM automatically selects important features based on their information gain, thereby eliminating subjectivity from prior variable selection and enabling the construction of an objective, data-driven prediction model, as previously reported [39].

To evaluate the model’s performance, we applied nested cross-validation [40], a validation method equivalent to external validation, to avoid overfitting and accurately evaluate the generalization performance. Specifically, stratified k-fold cross-validation with four outer folds and three inner folds was performed. The outer folds were used to evaluate the generalization performance of the model, whereas the inner folds were used to optimize the hyperparameters. For hyperparameter optimization, we used Bayesian optimization with Optuna (https://www.optuna.org/, accessed on 13 June 2025) to identify optimal parameters. Feature importance was extracted from the LightGBM models trained in each outer fold using gain (based on the improvement of the objective function) as an indicator. Feature importance was obtained for each model, and the average value across all folds was calculated to stably evaluate the contribution.

### 4.3. Evaluation Metrics

The predictive performance of the classification model was evaluated using information calculated from a mixed matrix containing the number of true positives (*TP*, compounds correctly predicted as positive), true negatives (*TN*, compounds correctly predicted as negative), false negatives (*FN*, positive compounds misclassified as negative), and false positives (*FP*, negative compounds misclassified as positive). The classification model was evaluated using the following five evaluation metrics [41]:

(1) *Sensitivity*: accuracy of predicting “positive” when the true result is positive.

(1) $$Sensitivity=\frac{TP}{TP+FN}$$

(2) *Specificity*: accuracy of predicting “negative” when the true result is negative.

(2) $$Specificity=\frac{TN}{TN+FN}$$

(3) *Balanced accuracy*: average sensitivity and specificity.

(3) $$Balanced\;accurancy=\frac{1}{2}(Sensitivity+Specificity)$$

(4) *MCC*: a measure for evaluating the classification accuracy of models for imbalanced datasets [42].

(4) $$MCC=\frac{\left(TP\times TN\right)-(FP\times FN)}{\sqrt{\left(TP+FP)(TP+FN)(TN+FP)(TN+FN\right)}}$$

(5) *AUC*: A graph presenting the performance of a classification model at all thresholds. This curve plots two parameters: sensitivity and 1 − specificity [43].

To determine the optimal cutoffs for TP, FN, TN, and FP, Youden’s index [44], which maximizes sensitivity (1 − specificity), was used. The cutoffs specific to the prediction model were standardized in drug prediction using the following equation:

(5) $$Xn={Xu}^{{-\log}_{c}2}$$

where we obtain Xn by normalizing the directly predicted value Xu, and c represents the cutoff for each prediction model.

### 4.4. FAERS Database

The FAERS database was accessed on the FDA’s official website [20], and data reported from the first quarter of 2004 to the third quarter of 2024 were used. Adverse events in the REAC table are registered on the basis of MedDRA basic terms (preferred terms), and the curation of drug names in DRUG was performed by Intage Healthcare Co., Ltd. (Tokyo, Japan). Identification numbers were assigned to cases, and each data table can be integrated. In the analysis, the DRUG and REAC tables were combined, and duplicates were removed.

### 4.5. Application of the Predictive Model to Drugs Listed in the FAERS Database

Of the 9400 drug names listed in the DRUG table of FAERS, we attempted to obtain SMILES notations by searching the PubChem database using the Python PubChem library (https://github.com/mcs07/pubchempy, accessed on 13 June 2025) [45,46]. We applied the PXR agonist prediction model constructed to the 5523 drug names for which the SMILES notation was successfully obtained. In order to ensure consistency in processing and reproducibility in descriptor calculations for thousands of datasets, we limited our analysis to drugs whose SMILES structures already existed on PubChem and did not convert SMILES from other formats. The model output probabilities were normalized according to the cutoff of each prediction model using Equation (5). Compounds with normalized scores of 0.7 or higher were defined as having PXR agonist activity. Additionally, among the drugs with experimental activity data from PXR assays, labels were corrected for the 161 compounds that did not match the model predictions based on experimental data. PCA was performed to visually evaluate the structural similarity between compounds derived from the Tox21 database and drugs listed in FAERS that were predicted to have PXR agonist activity. All 336 MOE descriptors used in the construction of the predictive model were used for PCA. After preprocessing, these descriptors were calculated by MOE based on the SMILES structures of each compound. PCA was performed on the compounds listed in Tox21, and the FAERS drugs were projected into the same principal component space using the principal component equations. The comparison of structural distributions was based on the relationship between principal components 1 and 2.

### 4.6. Relationship Between Drugs with Predicted PXR Agonist Activity and Adverse Events

Using the data table for analysis, we created a 2 × 2 contingency table (Table 2) and detected the signals of adverse events related to PXR agonist drugs. When cells containing 0 are present in a 2 × 2 contingency table, calculations cannot be performed, and when the frequency is extremely small, the accuracy of the estimation decreases. To correct this bias, we applied the Haldane–Anscombe correction by adding 0.5 to all cells [47]. The ROR and *p*-values for adverse events associated with drugs predicted to have PXR agonist activity were calculated using Fisher’s exact probability test. A relative evaluation was performed by detecting signals for adverse event terms represented by RORs. This unbalanced analysis method enabled the identification of cases in which many ADEs were reported [48]. Based on the calculated values, a volcano plot was created with the vertical axis representing −log*p* and the horizontal axis representing lnROR (Figure 4). The volcano plot was used to visually interpret the ADEs. Volcano plots are frequently used to understand trends in gene expression in microarray data analysis [49]. Adverse events with statistically significant associations with PXR agonist activity (lnROR > 1) and those *p* > 0.05 and more than 1000 reports were selected.

### 4.7. Statistical Analysis

Statistical analysis of adverse events (ROR, *p*-value, and 95% confidence intervals) was performed using a Python script (Python version 3.12.7). The volcano plots were created using JMP Pro 18.0.2 (SAS Institute Inc., Cary, NC, USA). The significance level was set at *p* < 0.05.

## 5. Conclusions

In this study, we applied a PXR agonist prediction model based on the Tox21 database to drugs in the FAERS database and conducted an inclusive analysis of the associations between adverse events and drugs predicted to have PXR agonist activity. The findings illustrated that terms related to cardiac disorders were more likely to be induced than other adverse events, suggesting that PXR agonist activity can contribute to cardiac-related adverse events. These findings highlight the need for early screening for PXR agonist activity in drug development risk assessment and post-marketing safety surveillance to predict and avoid cardiac side effects.

## Figures and Tables

**Table 1:** Distribution of adverse event terms deemed statistically significant by the SOC classification.

SOC Category	Significant Terms	Total Terms in SOC	Significant Term Rate (%)
Cardiac disorders	30	719	4.17
Product issues	5	203	2.46
Endocrine disorders	14	639	2.19
Blood and lymphatic system disorders	22	1165	1.89
Reproductive system and breast disorders	24	1350	1.78
Metabolism and nutrition disorders	16	918	1.74
Respiratory, thoracic and mediastinal disorders	22	1329	1.66
General disorders and administration site conditions	22	1426	1.54
Hepatobiliary disorders	6	490	1.22
Psychiatric disorders	11	908	1.21
Gastrointestinal disorders	22	1986	1.11
Immune system disorders	9	936	0.96
Renal and urinary disorders	6	826	0.73
Pregnancy, puerperium and perinatal conditions	5	700	0.71
Neoplasms benign, malignant and unspecified (incl. cysts and polyps)	15	2510	0.60
Musculoskeletal and connective tissue disorders	9	1562	0.58
Vascular disorders	11	1916	0.57
Nervous system disorders	13	2361	0.55
Injury, poisoning and procedural complications	14	2817	0.50
Infections and infestations	10	2353	0.42
Investigations	27	6407	0.42
Surgical and medical procedures	7	2607	0.27
Congenital, familial and genetic disorders	2	1859	0.11
Eye disorders	1	1196	0.08

**Table 2:** Cross-tabulation table and ROR formula.

	Adverse Event Present	Adverse Event Absent
PXR-Active Drug Exposure	a	b
Other Drug Exposure	c	d

ROR, reporting odds ratio [(a/b)/(c/d) = a × d/b × c].

## Data Availability

Data are contained within the article and Supplementary Materials.

## Supplementary Materials

The following supporting information can be downloaded at: https://www.mdpi.com/article/10.3390/ijms26157630/s1.
